# Identification, distribution, and hosts of *Meloidogyne* spp. infecting horticultural crops in Florida, USA with focus on *Meloidogyne enterolobii*

**DOI:** 10.2478/jofnem-2024-0042

**Published:** 2025-01-24

**Authors:** Gabrieli Riva, Janete A. Brito, Clemen de Oliveira, Marcus Marin, Mengyi Gu, Hung Xuan Bui, Johan Desaeger

**Affiliations:** Department of Entomology and Nematology, Gulf Coast Research and Education Center, University of Florida, Wimauma, FL, 33598, USA; Florida Department of Agriculture and Consumer Services, Division of Plant Industry, Nematology Section, P.O. Box 147100, Gainesville, FL 32614-7100, USA; Department of Plant Pathology, Gulf Coast Research and Education Center, University of Florida, Wimauma, FL, 33598, USA

**Keywords:** molecular identification, phylogeny, root-knot nematodes

## Abstract

Many root-knot nematode (RKN) species in the genus *Meloidogyne* occur in Florida, including *M. enterolobii*, a species able to overcome RKN resistance genes in many crops. The distribution of these nematodes in horticultural crops is not well known. A RKN survey was conducted in South and Central Florida aiming to: (i) identify RKN infecting vegetables, fruit, and other crops; (ii) document host plants; (iii) determine RKN distribution; and (iv) gain insight on the relatedness of *M. enterolobii* obtained in this study with other populations from the USA and other countries. A total of 304 soil and root samples were collected from 56 plant species cultivated in commercial vegetable and fruit farms, research farms, horticultural gardens, Asian vegetable farms, and natural landscapes in 12 counties. *Meloidogyne* species identification was performed using mitochondrial haplotype-based identification, species-specific primers, DNA sequencing and phylogenetic analysis. RKN were detected in 247 out of 304 (81.25%) root samples collected from September 2019 to January 2023. Five RKN species (*M. arenaria*, *M. enterolobii*, *M. hapla*, *M. incognita* and *M. javanica*) were identified. The most prevalent RKN were *M. incognita* and *M. enterolobii*, which were found in 25% of the samples. Less prevalent were *M. javanica*, found in 16%, and *M. arenaria* and *M. hapla*, found in 8% and 5% of samples, respectively. Mixed populations of *M. enterolobii* and *M. incognita* were found in 1% of the samples. Phylogenetic analysis showed low genetic variability among DNA sequences of *M. enterolobii* populations from Florida, other states in the USA, and other countries. New host records found in this study include: a worldwide host record, *Solanum capsicoides* (*M. enterolobii*); new US continental host records, *Vigna unguiculata* (*M. enterolobii*), *Opuntia cochenillifera* (mixed species – *M. enterolobii* and *M. incognita*). Additionally, new state host records found were *Cannabis sativa*, *Colocasia esculenta*, and *Lilium* sp. (*M. arenaria*), *Phaseolus vulgaris* (*M. enterolobii*), *Cucumis melo* (*M. hapla*), and *Lavandula angustifolia* and *Helianthus annuus* (*M. incognita*). These findings confirm the predominance of tropical RKN species, and especially of *M. enterolobii*, in Florida. and provide new insights into the distribution, prevalence, and hosts of RKN species in horticultural crops in Central and South Florida.

Florida ranks second in US vegetable sales, with its major vegetable production totaling $ 1.93 billion in 2022 (NASS, 2023). Most vegetable production in Florida occurs in the open field and on plastic-mulch raised beds in combination with drip irrigation.

Root-knot nematodes (RKN) (*Meloidogyne* spp.) top the chart of plant parasitic nematodes, with wide host ranges that span most cultivated crops, and are one of the most economically important plant pathogens in the U.S. and worldwide. The deep, fine sandy soils of Florida are very conducive for RKN, and these nematodes have been found damaging many economically important crops since the mid-1800s (Neal, 1889). RKN are among the most widespread plant-parasitic nematodes in Florida, with almost every vegetable crop grown in the state prone to RKN damage – particularly tomato, pepper, cucumber, squash, cantaloupe, watermelon, celery, lettuce, and potato (Neal, 1889; Desaeger, 2018). Currently, 17 species of RKN have been reported in Florida (Table 1). Among these species, *M. arenaria*, *M. enterolobii*, *M. hapla, M. haplanaria*, *M. incognita*, and *M. javanica* have been reported on horticultural crops during the past decade (Brito et al. 2008; 2010; Baidoo et al., 2016; Joseph et al., 2017; Smith et al., 2017).

**Table 1:** *Meloidogyne* spp. present in Florida.

***Meloidogyne* spp.**	**County**	**Year**	**References**
*M. arenaria*	Alachua	1888	Neal, 1889^a^; Chitwood, 1949
*M. artiellia* ^b^	Palm Beach	1986	Lehman, 2002
*M. christiei* ^c^	Seminole	1986	Golden and Kaplan, 1986
*M. cruciani*	Alachua	1986	Garcia-Martinez et al., 1982
*M. enterolobii* (= *M. mayaguensis*)	Dade	2002	Brito et al., 2002 ^d^
*M. floridensis*	Palm Beach Alachua	2004	Handoo et al., 2004
*M. graminicola*	Dade	2008	Brito et al., 2008
*M. graminis* (= *Hysoperine graminis*)	Polk	1959	Sledge and Golden, 1964^e^; Whitehead, 1968
*M. hapla*	Orange	1955	Lehman, 2002
*M. haplanaria*	Collier	2016	Joseph et al., 2016
*M. incognita*	Jefferson	1955	Lehman, 2002
*M. javanica*	Orange	1955	Lehman, 2002
*M. marylandi*	Marion	2012	Sekora et al., 2012
*M. megatyla* ^b^	Baker	1984	Lehman, 2002
*M. partityla*	Madison	2005	Crow et al., 2005
*M. spartinae* (= *Hypsoperine spartinae*)	Flagler	1958	Rau and Fassuliotis, 1965^f^; Whitehead, 1968
*M. thamesi*	Palm Beach	1952	Chitwood et al., 1952

a First found in 1888, with species description published in 1889.

b These records do not provide morphological descriptions, morphometrics or illustrations of the identified samples; therefore, their identity remain uncertain.

c Currently only reported in Florida, USA.

d Reported in the Continental USA, Palm Beach, and Dade Counties.

e First found in 1959. Nematode genus and species description were first published in 1964.

f First found in 1958, with species description published in 1965.

However, there is insufficient information on the distribution and prevalence of RKN species in Florida vegetable fields. This lack of information may be attributed to the common use of fumigant nematicides that make RKN almost undetectable in treated soil, the lack of females, and the almost exclusive presence in routine diagnostic samples of second-stage juveniles (J2) (which alone make RKN speciation difficult). Many of the high-value crop producers in Florida, including most vegetable growers, fumigate their fields before planting, which typically provides good control irrespective of the root-knot species present. This is the reason why these growers are generally not concerned about which RKN species are present in their fields. However, when integrated nematode management tactics are used, such as resistant cultivars, cover crops, crop rotations and biological control, it is more imperative to know the actual RKN species present in the field. This is especially important for *M. enterolobii*, as this species will overcome the current RKN resistance genes in many economic plant species (Carneiro et al., 2006; Brito et al., 2007; 2020; Cetintas et al., 2008; Kiewnick et al. 2009; Pinheiro et al., 2015).

As mentioned previously, the morphological identification of *Meloidogyne* species is very challenging and requires morphometric and morphological analyses of females and males as well as J2. The results of these analyses, however, are not reliable because of the variability of the morphology and measurements of RKN. The use of enzymatic analyses of females has provided a solid tool to validate identification of RKN made by morphological analyses. However, these enzymatic analyses can only be performed using females and specific enzymes, particularly esterase and malate dehydrogenase, for a correct identification.

Molecular methods have been demonstrated to be a valuable and practical approach to identifying RKN species, and they have been used to explore genetic diversity and population variations, as well as the evolutionary or taxonomic relationships of closely related species (Blok and Powers, 2009; Brito et al., 2016; Moore, 2020a; 2020b; Shao et al., 2020). DNA markers – including a region of variable size in *Meloidogyne* spp. between the mitochondrial COII and the large (16S) rRNA gene (Powers and Harris, 1993; Tigano et al., 2005); the small subunit 18S rRNA gene (De Ley et al., 2002; Tigano et al., 2005); the large subunit 28S D2-D3 expansion segments of the rRNA gene (Tenente et al., 2004); and the ITS of rRNA gene (Landa et al., 2008) – have been used for nematode species differentiation. However, the mitochondrial DNA (mtDNA) has proved to be much more consistent and robust for RKN species identification, particularly for the tropical species found infecting many plant species in different parts of the world (Devran and Sogut, 2009; McClure et al., 2012; Ye et at., 2015; Baidoo et al., 2016; Khanal et al., 2016).

The use of the molecular approach for the identification of RKN has become more common in recent years in Florida (Brito et al., 2004; 2008; 2016; 2020; Jeyaprakash et al., 2006; Tigano et al., 2010; Smith et al., 2015; Baidoo et al., 2016; Moore et al., 2020a; 2020b), but has been mostly limited to isolated findings. Molecular species information on the distribution of *Meloidogyne* spp. in Florida is still limited, especially in vegetable and fruit fields. The overall aim of this study was to learn more about the distribution, hosts and species of the RKN found in these fields in Central and South Florida, with particular focus on *M. enterolobii*, and acquire more insight into the usefulness of the molecular approach to identify RKN species in large-scale surveys. The specific objectives were to (i) identify RKN infecting vegetables, fruit, and other crops; (ii) document host plants; (iii) determine RKN distribution; (iv) gain insight on the relatedness of Florida *M. enterolobii* with other populations from the USA and other geographical areas. For this phylogenetic study, we used some of the DNA sequences newly obtained with COXII, which have shown to be of high diagnostic value in distinguishing *M. enterolobbii* from the other RKN species.

## Materials and Methods

*Sampling and processing:* A total of 304 root and soil samples from 56 different mostly fruit and vegetable crops were collected in 12 counties located in Central and South Florida from September 2019 to January 2023. The Global Positioning System (GPS) coordinates for each site were recorded using the GPS Tracks version 4.0.6 smartphone app (DM Software Solutions, LLC). Samples were collected from different cropping systems, including commercial vegetable farms; fruit and Asian vegetable farms; research farms; horticultural gardens; and natural landscapes. Samples were collected throughout the year and typically towards the end of the crop season, when symptoms, such as stunting, chlorosis and wilting, were most visible. A conical core sampler and shovel were used to take soil and root samples up to a depth of about 20 cm; the soil was collected with roots, with an effort to maintain their quality). The number of subsamples taken per sample depended on the location and size of the field and varied from a single sample in horticultural gardens and natural landscapes to three to ten plants per sample in commercial and research fields. Together with the roots, at least 500 cm^3^ of soil were collected and stored in a cooler (about 4 °C) for further nematode extraction, using a modified Baermann method (Regmi and Desaeger, 2020) when needed to confirm the absence of RKN in certain sample as stated below. Roots were carefully washed, and the presence of females and egg masses were checked under a dissecting microscope (Leica KL300 LED, Danaher Corporation, Washington, DC, USA). Three individual females per root system were hand-picked and used for RKN species identification. If no root galling, females, or egg masses were found, J2 were extracted from the soil sample, and in their absence, the sample was recorded as free from RKN. Plants were considered hosts of a given RKN species when root galls, females, and/or egg masses of that RKN were detected in their roots.

*DNA extraction, PCR amplification and sequencing:* DNA from each hand-picked female (N = 3) was extracted using the NaOH digestion method (Hübschen et al., 2004). Nematode species identification was conducted using nonspecific primer sets followed by species-specific primers (Table 2) and DNA sequencing of selected populations. DNA sequencing was only performed for populations that were previously identified as *M. enterolobii* and for a few other RKN populations where PCR identification gave inconsistent results. At least one nonspecific and species-specific primer set was used to identify the RKN present in each sample. PCR was performed using an Eppendorf Mastercycler Pro Thermal Cycler (Enfield, CT) in a 25 μl reaction volume consisting of 12.5 μl of 2 × Apex™ Taq Blue DNA Polymerase Master Mix (Genesee Scientific, San Diego, CA), 1 μl of DNA extract, 0.25 μl of each 10 μM primer stock, and 11 μl of sterile water. The PCR amplification conditions are reported in Table 3. The amplicons were separated by gel electrophoresis using a 1.2% Apex general purpose agarose gel (Genesee Scientific, El Cajon, CA), at 110 V for 60 min. Gels were stained with GelRed Nucleic Acid Stain (Biotium Inc., Hayward, CA), and DNA fragments were visualized under UV light using the Gel Doc EZ Imager (Bio-Rad Laboratories, Hercules, CA). DNA samples of *M. arenaria*, *M. enterolobii*, *M. incognita*, *M. javanica* (RKN collection, Division of Plant Industry, Florida Department of Agriculture and Consumer Services, Gainesville, FL) and *M. hapla* (RKN collection, Gulf Coast and Education Center University of Florida, Wimauma, FL) were used as positive controls for PCR in this study. As mentioned above, PCR products identified as positive for *M. enterolobii* were subject to DNA sequencing. For each *M. enterolobii*-positive sample, the best PCR product from at least one primer set was sent to Genewiz Company (South Plainfield, NJ, USA) for DNA purification and Sanger DNA sequencing. Newly obtained sequences were edited using Geneious Prime (version 2023.0.3, Biomatters, Ltd.) and deposited in the National Center for Biotechnology Information (NCBI) with the accession numbers indicated (Table 4).

**Table 2:** Primers used in this study.

**Code**	***Meloidogyne* spp.**	**Primer Sequence 5′-3′**	**Gene region**	**Reference**
Far	*M. arenaria*	TCGAGGGCATCTAATAAAGG	SCAR	Adams et al., 2007
Rar	*M. arenaria*	GGGCTGAATATTCAAAGGAA	SCAR	Adam et al., 2007
JMV-1	*M. hapla*	GGATGGCGTGCTTTCAAC	IGS - SCAR	Adam et al., 2007
JMV-2	*M. hapla*	AAAAATCCCCTCGAAAAATCCACC	IGS - SCAR	Adam et al., 2007
Me-F	*M. enterolobii*	AACTTTTGTGAAAGTGCCGCTG	IGS - rRNA	Long et al., 2006
Me-R	*M. enterolobii*	TCAGTTCAGGCAGGATCAACC	IGS - rRNA	Long et al., 2006
MI-F	*M. incognita*	GTGAGGATTCAGCTCCCCAG	SCAR	Meng et al., 2004
MI-R	*M. incognita*	ACGAGGAACATACTTCTCCGTCC	SCAR	Meng et al., 2004
Fjav	*M. javanica*	GGTGCGCGATTGAACTGAGC	SCAR	Zijlstra et al., 2000
Rjav	*M. javanica*	GGCCTTAACCGACAATTAGA	SCAR	Zijlstra et al., 2000
1108	Nonspecific	TACCTTTGACCAATCACGCT	COX2-l-rRNA	Powers and Harris, 1993
C2F3	Nonspecific	GGTCAATGTTCAGAAATTTGTGG	COX2-l-rRNA	Powers and Harris, 1993
D2A	Nonspecific	CAAGTACCGTGAGGGAAAGTTG	28S	Nunn, 1992
D3B	Nonspecific	TCGGAAGGAACCAGCTACTA	28S	Nunn, 1992
MORF	Nonspecific	ATCGGGGTTTAATAATGGG	IGS and tRNA-His	Hugall et al., 1994
MTHIS	Nonspecific	AAATTCAATTGAAATTAATAGC	IGS and tRNA-His	Hugall et al., 1994
NAD5-F2	Nonspecific	TATTTTTTGTTTGAGATATATTAG	NADH dehydrogenase subunit 5	Janssen et al., 2016
NAD5-R1	Nonspecific	CGTGAATCTTGATTTTCCATTTTT	NADH dehydrogenase subunit 5	Janssen et al., 2016
TRNAH	Nonspecific	TGAATTTTTTATTGTGATTAA	tRNA-His and l-rRNA	Stanton et al., 1997
MRH106	Nonspecific	AATTTCTAAAGACTTTTCTTAGT	tRNA-His and l-rRNA	Stanton et al., 1997

**Table 3:** The procedure of the PCR amplification used in this study.

**Primer**	**Response parameter (35 cycle)**				
	**Initial degeneration**	**Degeneration**	**Annealing**	**Extension**	**Final extension**
C2F3/1108	95 °C, 15 min	95 °C, 45 s	55 °C, 45 s	72 °C, 60 s	72 °C, 10 min
D2A/D3B	“	”	“	“	72 °C, 10 min
TRNAH/MRH106	“	95 °C, 30 s	50 °C, 30 s	68 °C, 60 s	68 °C, 10 min
MORF/MTHIS	“	“	“	“	68 °C, 10 min
Far/Rar	“	“	54 °C, 30 s	72 °C, 60 s	72 °C, 10 min
Fjav/Rjav	“	“	64 °C, 30 s	“	72 °C, 10 min
JMV1/JMV2	“	“	50 °C, 30 s	“	72 °C, 10 min
Me-F/Me-r	“	“	68 °C, 30 s	“	72 °C, 10 min
MI-F/MI-R	“	“	62 °C, 30 s	“	72 °C, 10 min
NAD5-F2/NAD5-R1	94 °C, 2 min	94 °C, 60 s	45 °C, 60 s	72 °C, 90 s	72 °C, 10 min

**Table 4:** *Meloidogyne* species and GenBank accession numbers of the newly DNA sequences obtained in the present study.

**Sample No.**	**Location (County)**	**Plant host**	**RKN species**	**Gene region**	**Accession number**	**References**
FL 21	Hillsborough	*Cucumis sativus*	*M. arenaria*	NADH dehydrogenase subunit 5	OR043670	This study
FL 22	Hillsborough	*Solanum lycopersicum*	*M. arenaria*	NADH dehydrogenase subunit 5	OR043669	This study
FL 2	Manatee	*Solanum lycopersicum*	*M. enterolobii*	COX2 - l-rRNA	OQ680023	This study
FL 3	Hillsborough	*Luffa cylindrica*	*M. enterolobii*	COX2 - l-rRNA	OQ680018	This study
FL 4	Hillsborough	*Ipomoea batatas*	*M. enterolobii*	COX2 - l-rRNA	OQ680019	This study
FL 11	Manatee	*Solanum lycopersicum*	*M. enterolobii*	COX2 - l-rRNA	OQ835723	This study
FL 13	Hendry	*Capsicum annuum*	*M. enterolobii*	COX2 - l-rRNA	OQ680020	This study
FL 14	Manatee	*Cucumis sativus*	*M. enterolobii*	COX2 - l-rRNA	OQ680021	This study
Fl 15	Hillsborough	*Capsicum annuum*	*M. enterolobii*	COX2 - l-rRNA	OQ680022	This study
FL 16	Hillsborough	*Capsicum annuum*	*M. enterolobii*	COX2 - l-rRNA	OR161827	This study
FL 13	Hendry	*Capsicum annuum*	*M. enterolobii*	28S rRNA	OQ508955	This study
FL 4	Hillsborough	*Ipomoea batatas*	*M. enterolobii*	tRNA-His and l-rRNA	OQ680025	This study
FL 11	Manatee	*Solanum lycopersicum*	*M. enterolobii*	tRNA-His and l-rRNA	OQ835724	This study
FL 13	Hendry	*Capsicum annuum*	*M. enterolobii*	tRNA-His and l-rRNA	OQ680026	This study
FL 16	Hillsborough	*Capsicum annuum*	*M. enterolobii*	tRNA-His and l-rRNA	OQ680027	This study
Fl 17	Hillsborough	*Nopalea cochenillifera*	*M. enterolobii*	tRNA-His and l-rRNA	OQ835727	This study
FL 18	Palm Beach	*Capsicum annuum*	*M. enterolobii*	tRNA-His and l-rRNA	OQ835725	This study
FL 19	Palm Beach	*Capsicum annuum*	*M. enterolobii*	tRNA-His and l-rRNA	OQ835726	This study
FL 20	Hillsborough	*Cucurbita pepo*	*M. enterolobii*	tRNA-His and l-rRNA	OQ680028	This study
FL 2	Manatee	*Solanum lycopersicum*	*M. enterolobii*	tRNA-His and l-rRNA	OQ680024	This study
FL 3	Hillsborough	*Luffa cylindrica*	*M. enterolobii*	tRNA-His and l-rRNA	OR161828	This study
FL 24	Miami Dade	*Lablab purpureus*	*M. incognit*a	NADH dehydrogenase subunit 5	OR043668	This study
FL 25	Manatee	*Solanum lycopersicum*	*M. incognita*	NADH dehydrogenase subunit 5	OR033166	This study
FL 26	Manatee	*Solanum lycopersicum*	*M. incognita*	NADH dehydrogenase subunit 5	OR033167	This study
FL 27	Manatee	*Solanum lycopersicum*	*M. incognita*	NADH dehydrogenase subunit 5	OR043667	This study
FL 28	Palm Beach	*Cucumis sativus*	*M. incognita*	NADH dehydrogenase subunit 5	OR033164	This study
FL 29	Palm Beach	*Solanum lycopersicum*	*M. incognita*	NADH dehydrogenase subunit 5	OR033168	This study
FL 30	Palm Beach	*Abelmoschus esculentus*	*M. incognita*	NADH dehydrogenase subunit 5	OR033163	This study
FL 31	Hillsborough	*Solanum lycopersicum*	*M. incognita*	NADH dehydrogenase subunit 5	OR033165	This study

Prior to phylogeny inference, sequences were aligned and trimmed to remove ambiguously aligned residues using Geneious Prime. Phylogenetic assumptions, stationarity (constant nucleotide frequencies over time), and homogeneity (constant substitution rates over time) were assessed using matched-pairs tests of symmetry implemented in IQ-TREE (version 2.1.2; Minh et al., 2020; Naser-Khdour et al., 2019). The best-fit substitution model was then determined by ModelFinder for each partition according to Bayesian Information Criterion (BIC) (Kalyaanamoorthy et al., 2017), resulting in HKY + F + G4 for both COX2-l-rRNA and l-rRNA genes. Phylogenetic tree construction, based on the two nonspecific primer sets mentioned above, was performed using MrBayes (version 3.2.7; Ronquist et al., 2012) with the following settings: two independent runs, one million generations, sampling every 1,000 generations, and discarding the first 25% of trees as burn-in. The remaining trees were used for generating a 50% majority-rule consensus tree with posterior probabilities for each node, and nodal support was estimated based on 1,000 standard bootstrap replicates. The phylogenetic trees were rooted to the outgroup species of each gene, visualized, and edited using FigTree (version 1.4.4; http://tree.bio.ed.ac.uk/software/figtree/) and Inkscape (version 1.0.1; https://inkscape.org/), respectively. The sequence alignments used in this study to build the phylogenetic trees can be found in the GenBank accession numbers listed on each phylogenetic tree.

For the phylogenetic tree based on DNA fragments of the COX2-l-rRNA gene from C2F3/1108 primer, a total of 44 sequences –36 from GenBank and 8 of *M. enterolobii* – were selected for this study. These sequences were from 13 countries (Australia, Brazil, Burkina Faso, China, Kenya, Mexico, Portugal, Italy, Ivory Coast, Japan, Slovenia, South Africa, and USA). The USA sequences were from six states (Arkansas, Florida, Georgia, Nebraska, North Carolina, and South Carolina). For the phylogenetic tree based on the tRNA-His and l-rRNA gene from TRNAH/MRH106 primers, a total of 36 sequences – 26 from GenBank and 10 of *M. enterolobii* – were selected for this study. These sequences were from five countries (Brazil, China, Kenya, Mexico, and USA). The USA sequences were from four states (Georgia, Georgia, North Carolina, and South Carolina).

*Survey data collection and display:* Data concerning the RKN species identified were listed according to their geographical location, plant host, and the management practices of the field operations sampled.

## Results

The findings of our survey have agronomic significance for agricultural specialists at local and national level. They enrich their knowledge about the host preference of the RKN identified in the vegetable crop systems adopted in Florida operations. The survey data are therefore presented in different categories to facilitate their interpretation by growers, agricultural specialists and extension agents in Florida and in the USA.

**Figure 1:**
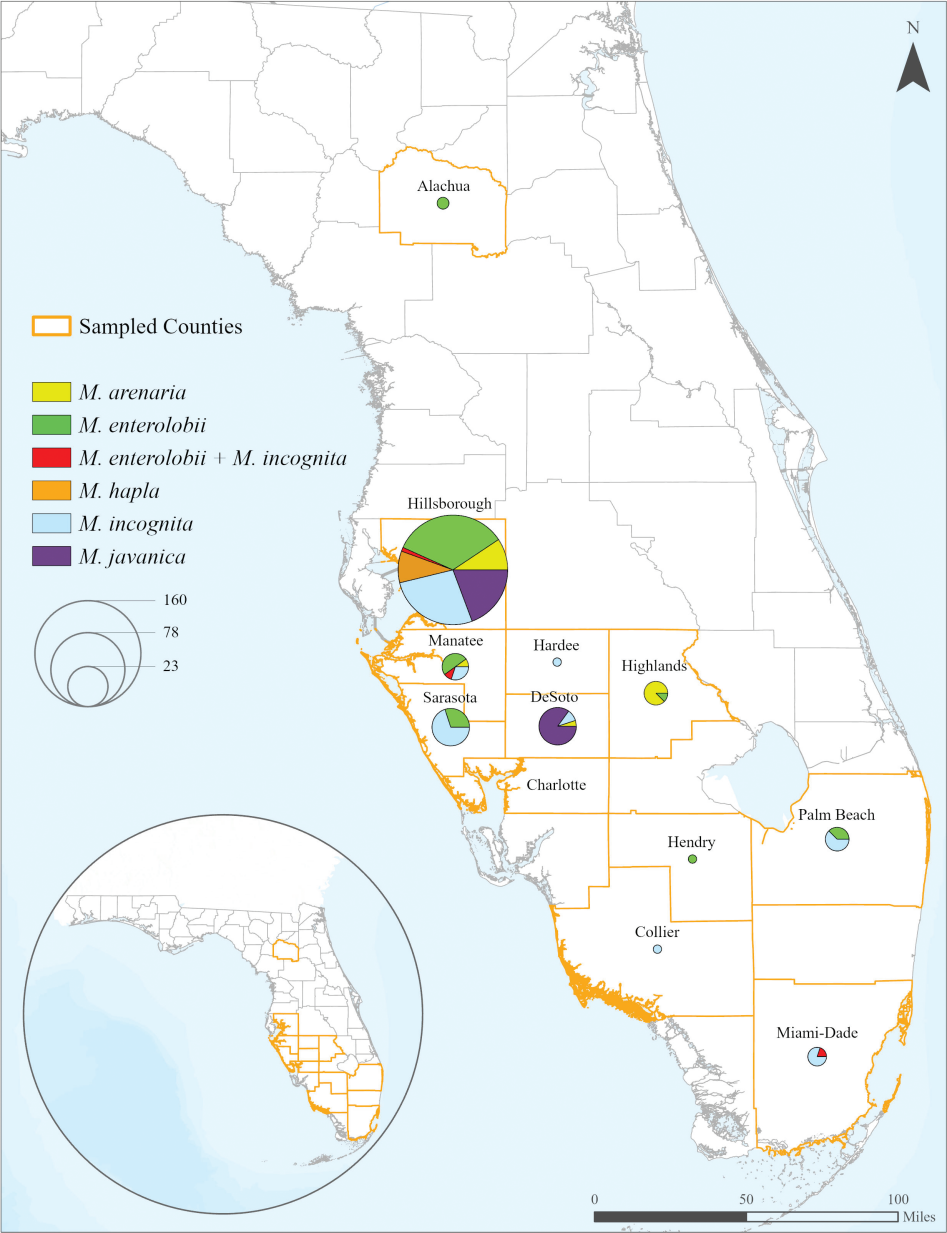
Florida's map showing the 12 counties sampled, distribution, and prevalence of each *Meloidogyne* species identified in this study. Out of 304 samples collected, 247 were positive for root-knot nematodes. Counties sampled and number of samples positive for *Meloidogyne* spp. were Alachua (2), Collier (1), DeSoto (20), Hardee (1), Hendry (1), Highlands (8), Hillsborough (171), Manatee (10), Miami-Dade (5), Palm Beach (8), and Sarasota (20). No root-knot nematodes were found in the samples collected in Charlotte County. Map was made using ArcGIS Prop software (Esri, Redlands, CA).

**Figure 2:**
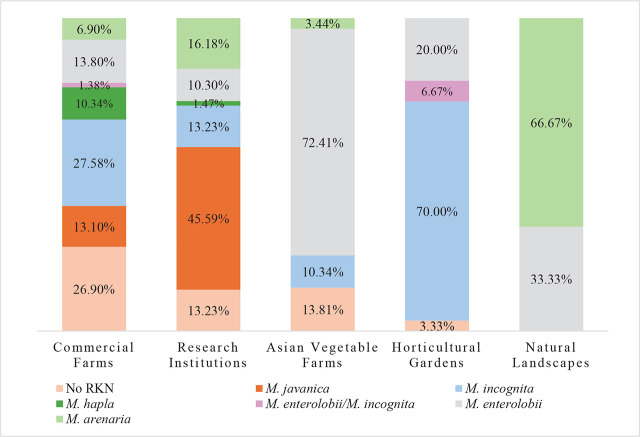
Distribution of *Meloidogyne* species across different cropping systems found in this study.

*By geography:* A total of 304 root and soil samples were collected from 12 counties (Alachua [2], Charlotte [20], Collier [10], DeSoto [26], Hardee [1], Hendry [3], Highlands [8], Hillsborough [176], Manatee [11], Miami Dade [14], Palm Beach [13] and, Sarasota [20]) in Central and South Florida from September of 2019 to January of 2023 (Fig. 1). Samples were collected from different locations, including commercial vegetable farms, fruit and Asian vegetable farms, research farms, horticultural gardens, and natural landscapes (Fig. 2). At least one RKN species was detected in every county sampled, except Charlotte (Fig. 1). RKN species were detected in 247 (81.25%) of the 304 samples collected. At least 741 females were subject to molecular analysis (247 × 3).

**Figure 3:**
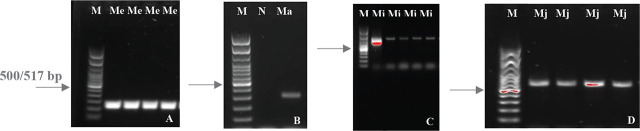
PCR amplification product generated using species-specific primer sets. A) Me = *Meloidogyne enterolobii* genomic DNA amplified using Me-F/Me-R primer set; B) Ma = *Meloidogyne arenaria* genomic DNA amplified using Far/Rar primer set; C) Mi = *Meloidogyne incognita* genomic DNA amplified using MI-F/MI-R primer set; D) Mj = *Meloidogyne javanica* genomic DNA amplified using Fjav/Rjav primer set; N = Water; M = 100-bp DNA ladder (New England Biolabs, MA), with the arrow indicating the position of the 500/517 bp band.

Based on the molecular analyses, including species-specific primers (Fig. 3) and DNA sequencing a total of five RKN species were identified in this study: *M. arenaria*, *M. enterolobii*, *M. hapla*, *M. incognita*, and *M. javanica*. *M. incognita* and *M. enterolobii* were the two most prevalent species, with each present in 76 (25.00%) samples. *M. javanica* was identified in 50 samples (16.44%), followed by *M. arenaria* with 25 samples (8.22%) and *M. hapla* with 16 samples (5.27%). Mixed populations of *M. enterolobii* and *M. incognita* were identified in four samples (1.31%). Hillsborough County was the most intensively sampled, with 171 samples positive for RKN. Other counties sampled where RKN was detected on at least one sample were Alachua (2), Collier (1), DeSoto (20), Hardee (1), Hendry (1), Highlands (8), Manatee (10), Miami-Dade (5), Palm Beach (8), and Sarasota (20) (Fig. 1). *M. enterolobii* was found in seven counties: Alachua, Hendry, Highlands, Hillsborough, Manatee, Palm Beach, and Sarasota. This represents 58% of the sampled counties and 63% of the counties found to be positive for RKN (Fig. 1).

*By growing conditions and farm type:* Most samples were collected from commercial vegetable, fruit, and ornamental farms, representing a total of 145 samples (47.70%) with 106 positive samples for RKN. The crops sampled were tomato (46), pepper (23), strawberry (11), squash (7), cantaloupe (6), caladium (6), okra (5), cucumber (4), eggplant (3), guava (3), cherry tomato (3), boniato (3), parsley (3), sugar cane (3), sugarbeet (2), watermelon (2), bean (2), Caesar weed (1), corn (1), elephant ear (1), indigo (1), jackfruit (1), lily (1), luffa (1), Italian parsley (1), and peach (1) (Table 5). Five species of RKN nematodes were identified from these farms: *M. incognita*, *M. enterolobii*, *M. javanica*, *M. hapla*, *M. arenaria* and mixed populations of *M. enterolobii* and *M. incognita*. These represented 40 (27.58%), 20 (13.80%), 19 (13.10%), 15 (10.34%), 10 (6.90%) and 2 (1.38%) samples, respectively (Fig. 2).

**Table 5:** *Meloidogyne* species found infecting different plant species in this study.

**Crop**	**Scientific name**	**Family**	**Total number of samples^a^**	***M. arenaria***	***M. enterolobii***	***M. incognita***	***M. javanica***	***M. hapla***	***M. enterolobii*/*M. incognita***
Tomato	*Solanum lycopersicum*	Solanaceae	67 (9)	4	6	22	25	0	1
Pepper	*Capsicum annuum*	Solanaceae	28 (1)	0	16	10	0	1	0
Caladium	*Caladium bicolor*	Araceae	14	14	0	0	0	0	0
Strawberry	*Fragaria × ananassa*	Rosaceae	14 (1)	0	0	0	2	11	0
Sweet potato	*Ipomoea batatas*	Convolvulaceae	14	0	14	0	0	0	0
Cucumber	*Cucumis sativus*	Cucurbitaceae	13	1	1	8	3	0	0
Cowpea	*Vigna unguiculata*	Fabaceae	11	0	1	0	10	0	0
Okra	*Abelmoschus esculentus*	Liliaceae	10 (1)	0	1	6	1	0	1
Squash	*Cucurbita pepo*	Cucurbitaceae	10 (1)	0	1	7	1	0	0
Luffa	*Luffa cylindrica*	Cucurbitaceae	9 (1)	0	7	1	0	0	0
Pumpkin	*Cucurbita pepo*	Cucurbitaceae	8	0	6	1	0	0	1
Basil	*Ocimum basilicum*	Lamiaceae	7	0	7	0	0	0	0
Cantaloupe	*Cucumis melo*	Cucurbitaceae	7	0	0	1	2	4	0
Eggplant	*Solanum melongena*	Solanaceae	6 (2)	0	4	0	0	0	0
Lettuce	*Lactuca sativa*	Asteraceae	6 (3)	0	0	3	0	0	0
Bean	*Phaseolus vulgaris L.*	Fabaceae	4 (2)	0	1	1	0	0	0
Cherry tomato	*Solanum lycopersicum*	Solanaceae	4	0	0	4	0	0	0
Lablab bean	*Lablab purpureus*	Fabaceae	4 (3)	0	0	1	0	0	0
Sugar cane	*Saccharum officinarum*	Poaceae	4 (4)	0	0	0	0	0	0
Amaranth	*Amaranthus sp.*	Amaranthaceae	3	0	0	3	0	0	0
Boniato	*Ipomoea batatas*	Convolvulaceae	3	0	0	0	0	0	0
Corn	*Zea mays*	Poaceae	3 (3)	0	0	0	0	0	0
Guava	*Psidium guajava*	Myrtaceae	3 (3)	0	0	0	0	0	0
Malabar spinach	*Basella alba*	Basellaceae	3	0	2	1	0	0	0
Parsley	*Petroselinum crispum*	Apiaceae	3 (3)	0	0	0	0	0	0
Watermelon	*Citrullus lanatus*	Cucurbitaceae	3 (2)	0	0	0	1	0	0
Artichoke	*Cynara cardunculus*	Asteraceae	2	0	0	1	1	0	0
Bok choy	*Brassica rapa*	Brassicaceae	2	0	0	1	0	0	0
Ginger	*Zingiber officinale*	Zingiberaceae	2	2	0	0	0	0	0
Golden Egg	*Solanum macrocarpon*	Solanaceae	2	0	2	0	0	0	0
Hemp	*Cannabis sativa*	Cannabaceae	2	2	0	0	0	0	0
Indigo	*Indigofera tinctoria*	Fabaceae	2 (1)	0	0	0	1	0	0
Jute	*Corchorus olitorius*	Tiliaceae	2	0	2	0	0	0	0
Perilla	*Perilla frutescens*	Lamiaceae	2	0	2	0	0	0	0
Radish	*Raphanus sativus*	Brassicaceae	2 (1)	0	0	0	1	0	0
Sugar beet	*Beta vulgaris*	Amaranthaceae	2	0	2	0	0	0	0
Sunflower	*Helianthus annuus*	Asteraceae	2 (1)	0	0	1	0	0	0
Thai basil	*Ocimum basilicum*	Lamiaceae	2	0	0	1	1	0	0
Cactus	*Nopalea cochenillifera*	Cactaceae	1	0	0	0	0	0	1
Caesar Weed	*Urena lobata*	Malvaceae	1(1)	0	0	0	0	0	0
Cauliflower	*Brassica oleracea*	Brassicaceae	1 (1)	0	0	0	0	0	0
Chrysanthemum	*Chrysanthemum indicum*	Asteraceae	1 (1)	0	0	0	0	0	0
Coriander	*Coriandrum sativum*	Apiaceae	1	0	0	1	0	0	0
Elephant ear	*Colocasia esculenta*	Araceae	1	1	0	0	0	0	0
Italian Parsley	*Petroselinum crispum*	Apiaceae	1 (1)	0	0	0	0	0	0
Jackfruit	*Artocarpus heterophyllus*	Moraceae	1 (1)	0	0	0	0	0	0
Lavender	*Lavandula angustifolia*	Lamiaceae	1	0	0	1	0	0	0
Leek	*Allium ampeloprasum*	Amaryllidaceae	1 (1)	0	0	0	0	0	0
Lily	*Lilium*	Lamiaceae	1	0	1	0	0	0	0
Mustard	*Brassica nigra*	Brassicaceae	1	0	1	0	0	0	0
Napa cabbage	*Brassica rapa*	Brassicaceae	1	0	0	1	0	0	0
Peach	*Prunus persica*	Rosaceae	1 (1)	0	0	0	0	0	0
Turnip	*Brassica rapa*	Brassicaceae	1	0	0	1	0	0	0
Water spinach	*Ipomoea aquatica*	Convolvulaceae	1	0	0	1	0	0	0
Wild tomato	*Solanum capsicoides*	Solanaceae	1	0	1	0	0	0	0
Zucchini	Cucurbita pepo	Cucurbitaceae	1 (1)	0	0	0	0	0	0

a Number of samples that were collected from each of the crops, with the number between parentheses representing the number of sample negative for *Meloidogyne* species infection.

Asian vegetable farms represented 58 samples (19.07%), with 50 of them positive for RKN. Three RKN species were detected with *M. enterolobii* as the predominant species, accounting for 42 samples (72.41%), followed by *M. incognita* with 6 samples (10.34%), and *M. arenaria*, with two samples (3.44%) (Fig. 2). The crops sampled were sweet potato (14), luffa (8), basil (7), pumpkin (7), perilla (2), Malabar spinach (2), amaranth (2), ginger (2), golden egg (2), jute (2), Thai basil (1), water spinach (1), radish (1), Napa cabbage (1), lettuce (1), leek (1), eggplant (1), chrysanthemum (1), cauliflower (1), and bok choy (1) (Table 5).

A total of 68 samples (22.37%) represented research institutions, including private companies and University of Florida Research Centers, with 59 positive samples for RKN. Five species of RKN were detected, with *M. javanica* representing 31 positive samples (45.59%), followed by *M. arenaria*, *M. incognita*, *M. enterolobii* and *M. hapla*, representing 11 (16.18%), 9 (13.23%), 7 (10.30%) of samples, and one (1.48%) positive sample, respectively (Fig. 2). The crops sampled were tomato (15), cowpea (10), caladium (9), cucumber (8), strawberry (3), squash (2), okra (2), pepper (2), squash (2), artichoke (2), corn (2), zucchini (1), watermelon (1), Thai basil (1), sunflower (1), sugar cane (1), radish (1), lettuce (1), lavender (1), indigo (1), eggplant (1), and cantaloupe (1) (Table 5).

A total of 30 (9.87%) samples were collected from horticultural gardens, with 29 positive samples for RKNs. Only two RKN species were found, with *M. incognita* the most common, being found in 21 (70.00%) of the samples; *M. enterolobii* in six of the samples (20.00%); and mixed populations of *M. incognita* and *M. enterolobii* in two of the samples (6.67%) (Fig. 2). The crops sampled were tomato (6), lettuce (4), okra (3), pepper (3), sunflower (1), squash (1), pumpkin (1), mustard (1), Malabar spinach (1), eggplant (1), cucumber (1), cowpea (1), coriander (1), cherry tomato (1), cactus (1), bok choy (1), and amaranth (1) (Table 5). Three samples were collected from natural areas; two samples were from hemp (*Cannabis sativa*) at a private home and were positive for *M. arenaria*; and one sample was collected from a wild tomato (*Solanum capsicoides*) in a natural preserve and was positive for *M. enterolobii* (Fig. 2).

**Figure 4:**
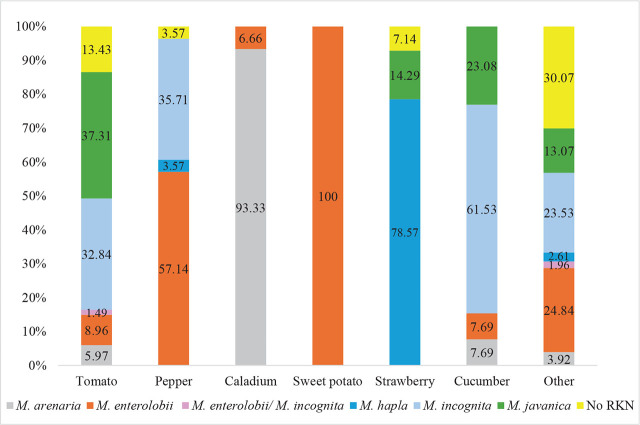
Distribution of *Meloidogyne* species by crop found in this study.

*By crop and plant species*: A total of 56, crops belonging to 22 different families, were sampled (Table 5). Tomato was the most-sampled crop, followed by pepper, caladium, sweet potato, and strawberry. Among the plant families, Solanaceae had 108 samples, followed by Cucurbitaceae (51), Fabaceae (21), Convolvulaceae (18), and Araceae (16) (Fig. 4, Table 5). *M. enterolobii* was found infecting 20 different plant species, with pepper (16), sweet potato (14), basil (7), luffa (7), pumpkin (6), and tomato (6) being the most common hosts. For comparison, *M. incognita* was found on 21 different plant species, *M. javanica* on 12, *M. arenaria* on six and *M. hapla* on three (Table 5).

Several new host records for *M. enterolobii* were found in this study: new worldwide host records of *M. enterolobii* infecting wild tomato (*Solanum capsicoides*); new continental USA host records of *M. enterolobii* infecting cowpea (*Vigna unguiculata*) and cactus (*Nopalea cochenillifera*); and new Florida host records of *M. enterolobii* infecting bean (*Phaseolus vulgaris* L.). There were also new state host records for Florida of *M. arenaria* infecting hemp (*Cannabis sativa*); elephant ear (*Colocasia esculenta*); and lily (*Lilium* sp.), *M. hapla* infecting cantaloupe (*Cucumis melo*), and *M. incognita* infecting lavender (*Lavandula angustifolia*) and sunflower (*Helianthus annuus*).

*Phylogenetic study: Meloidogyne enterolobii* sequences obtained from this study formed a monophyletic clade with other *M. enterolobii* populations with 100% support. All selected sequences from this study and other locations collected in the US and other parts of the world were used for comparisons grouped together, presenting genetic similarities among them.

**Figure 5:**
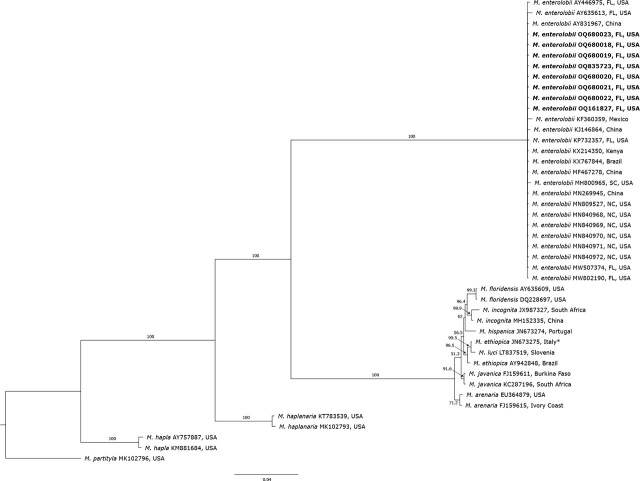
Bayesian 50% majority-rule consensus tree inferred from mitochondrial DNA DNA fragment of cytochrome c oxidase subunit II to large subunit 16S rRNA genes from C2F3/1108 primer set. Populations of *Meloidogyne enterolobii* obtained in this study are represented in bold. GenBank accession number proceed species and origin. ^*^Recently reclassified as *Meloidogyne luci* (Stare et al., 2017).

The phylogenetic tree, based on DNA fragments of the COX2-l-rRNA gene from C2F3/1108 primer set, placed RKN populations into five distinct groups (Fig. 5). The RKN populations clustered in five distinct clades containing sequences of 11 RKN species. In this tree, *M. enterolobii*, *M. hapla*, *M. haplanaria* and *M. partityla* showed genetic variation and clustered in four different clades. *M. arenaria*, *M. ethiopica*, *M. floridensis*, *M. hispanica*, *M. incognita*, *M. javanica* and *M. luci* clustered together in another separate clade, presenting genetic similarities among those species.

**Figure 6:**
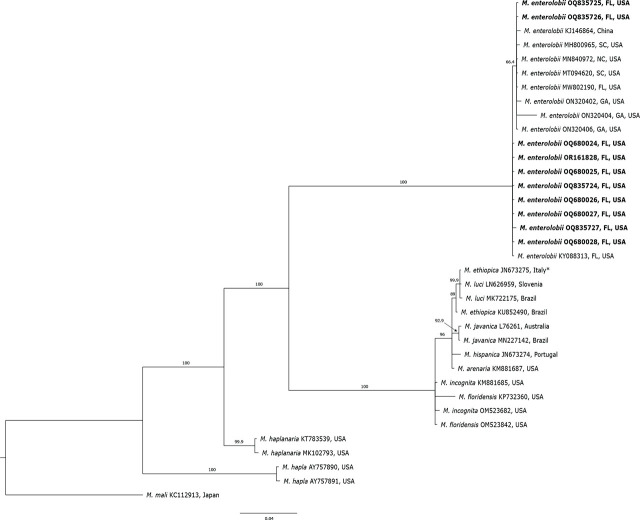
Bayesian 50% majority-rule consensus tree inferred from mitochondrial DNA fragment of partial l-rRNA gene from TRNAH/MRH106 primer set. *Meloidogyne enterolobii* from this study are represented in bold. Branch lengths represent means of posterior distribution. GenBank accession number precedes species and origin. ^*^Recently reclassified as *Meloidogyne luci* (Stare et al., 2017).

In the phylogenetic tree based on the tRNA-His and l-rRNA gene from TRNAH/MRH106 primer set, the RKN populations clustered in five distinct clades (Fig. 6) containing sequences of 11 RKN species. *Meloidogyne enterolobii*, *M. hapla*, *M. haplanaria* and *M. mali* clustered in four separate and distinct clades. *M. arenaria*, *M. ethiopica*, *M. floridensis*, *M. hispanica*, *M. incognita*, *M. javanica* and *M. luci* clustered in another separate clade, presenting genetic similarities among those species. The clade with *M. enterolobii* sequences contained a subclade with 66% support. In this subclade, two *M. enterolobii* isolates (OQ835725 and OQ835726) from this study clustered together with other isolates (KJ146864, MH800965, MN840972, MT094620, MW802190, ON320402, ON320404 and ON320406) from China, Florida, Georgia, North Carolina, and South Carolina.

## Discussion

Root-knot nematodes find perfect conditions in Florida and have been a menace to vegetable and specialty crop growers in the state since the early days of agriculture (Neal, 1889; Desaeger, 2021). Since the first report of *M. enterolobii* in Florida (Brito et al., 2002; 2004), two *Meloidogyne* surveys were carried out in the state (Brito et al. 2008, 2010). Both studies used isozyme analysis, mainly esterase (EST) and malate dehydrogenase (MDH) phenotypes, to characterize and identify the species of *Meloidogyne* present. In both these surveys, the most common RKN species found were *M. arenaria*, *M. incognita* and *M. javanica*, with *M. enterolobii* the most frequently found in ornamental plants. Many ornamental plants have been reported to be susceptible to *M. enterolobii*, and it is becoming clear that infected ornamental plants, during planting, are one of the major pathways of introducing and spreading *M. enterolobii* among several parts of the world (Moore et al. 2020a; 2020b; EPPO, 2023; de Heij, 2023).

In the southern US, *M. incognita* is the most identified RKN species in commercial fields (Schwarz et al., 2020; Marquez and Hajihassani, 2023; Faske et al., 2023). Our findings confirm the prevalence of *M. incognita* as the most common RKN species in Florida, but they also (and maybe surprisingly) confirm that *M. enterolobii* is just as prevalent. *M. enterolobii* was identified in 20 different crops, from large commercial fields to small community gardens. Several new host records for *M. enterolobii* were found in this study (as well as for other RKN species), confirming, and further expanding the already extremely broad host range that has been reported from ornamental, horticultural and agronomic crops, as well as turfgrass and weed plants, in previous studies from Florida (Brito et al., 2007, 2010; Baidoo et al., 2016; Joseph et al., 2016; Kaur et al., 2007).

In Asian vegetable farms, RKN, especially *M. enterolobii*, were very prevalent, often in high numbers. Most of these farms grow crops year-round, providing a continuous food supply to the nematodes, as most crops are good hosts to many RKN species (Bui et al, 2022a; 2022b). Nematode management is non-existent in these farms, and farmers generally have no knowledge about RKN or other nematodes. Due to language and cultural barriers (most farmers are of Vietnamese origin), Asian vegetable farms have had limited access to pest management information in general. It is quite likely that RKN, including *M. enterolobii*, has been spread across these farms with infested plant material, such as ginger rhizomes and sweetpotato tubers.

*Meloidogyne arenaria* was found in hemp, tomato, and cucumber, but was especially common in caladium, a very valuable ornamental crop that is grown for its tubers. Caladium has been reported to be susceptible to the three major RKN species, as well as to *M. enterolobii* and *M. floridensis* (Kokalis-Burelle et al., 2017). Additionally, caladium has a long growing period of more than six months, and RKN is the most important pathogen infecting this crop, especially when grown in sandy soil (Gu and Desaeger, 2021). Although hot water treatment, by soaking the tubers in water at 43° C for two hours, is considered an effective procedure to sanitize caladium tubers from RKN and Pythium, it has been observed that some nematodes are likely to survive (Gu and Desaeger, 2021), possibly (just like ginger in Asian vegetable farms) facilitating the spread of *M. arenaria* across caladium fields with tubers infested by nematodes that escaped the treatment.

*Meloidogyne javanica* was found on 12 different plants and was the most common RKN species on tomato. *M. javanica* was also detected infecting a specific strawberry cultivar, Winterstar, but it did not infect any of six other strawberry cultivars that were planted together with this cultivar (Oliveira et al., 2023). This was the first report of *M. javanica* infesting strawberry in Florida, where *M. hapla* has been the most reported RKN species on this crop. This has been confirmed by the findings in this study and previous field observations conducted by Oliveira (2022), indicating that *M. hapla* is being introduced into Florida fields with nematode-infested strawberry propagative material imported from northern states and Canada. Every year, more than 100 million strawberry transplants carrying the nematode are imported into Florida strawberry operations. *Meloidogyne hapla* can cause some late-season damage to Florida strawberry, but it can also reproduce rapidly on double- or relay-cropped vegetables grown soon after strawberry, preventing their establishment and severely stunting their growth (Desaeger, 2018; Khanal and Desaeger, 2020).

Significant regional differences in the prevalence of RKN throughout the state's vegetable area were noted. In the drier deep sand soils in west-central Florida, RKN has been long reported to be widely distributed (Neal, 1889), and visible crop damage was common in this area during our survey. In the Everglades agricultural region in south Florida (sections of Collier, Hendry, Palm Beach, and Miami-Dade counties) which has large areas of organic “muck” soils and a naturally high water table, vegetable crops rarely showed nematode damage, and RKN were also much more sporadic. The high water table and seasonal flooding of some of these fields, as well as the high organic content of the muck soils, naturally suppress RKN. Flooding has been used to control RKN since the 1930s (Brown, 1933), and organic soils are well-known to often provide excellent natural suppression of many plant-parasitic nematodes.

*Meloidogyne enterolobii* sequences from this study formed a monophyletic clade with other *M. enterolobii* populations with 100% support. The phylogenetic analysis of 12 different *M. enterolobii* isolates obtained from this study and 24 isolates of *M. enterolobii* from the USA and other countries clearly separated *M. enterolobii* from other RKN species and revealed low variability amongst them, clustering the isolates in one clade. This confirms the results of other studies from Brazil, South Africa and China (Tigano et al., 2010; Onkendi and Moleleki, 2013; Rashidifard et al., 2018, Shao et al., 2020). The lack of variation among *M. enterolobii* species can be explained by their mode of reproduction through mitotic parthenogenesis, and could also indicate possible transportation or movement of infected plant material and/or infested soil around the world. *M. enterolobii* has been intercepted in several European countries, including the Netherlands, Germany, United Kingdom, several times in plant material imported from Asia, South America, and Africa, including Cactaceae, *Syngonium* sp., *Ficus* sp., *Ligustrum* sp., *Brachychiton* sp., and *Rosa* sp. (EPPO, 2023; de Heij et al., 2023). While the *M. enterolobii* sequences here presented a lack of genetic diversity, it is important to point out the low number of analyses (< 100 samples), as well as the fact that phylogenetic trees based on l-rRNA indicated little variability among the isolates and placed the *M. enterolobii* isolates into a subclade with 66% support. Further studies analyzing more sequences are necessary to keep investigating possible genetic variation among populations found infecting different plant species in the USA and other counties.

This study confirmed the prevalence of RKN in South and Central Florida and revealed that *M. incognita* and *M. enterolobii* are the most common species in these parts of the state. On the other hand, *M. enterolobii* reports from North Florida are few, which may be related to the difference in crops grown there (Z. Grabau, UF, pers. comm.). Peanut (*Arachis hypogeae*) and corn (*Zea mays*), which are both non-hosts or poor hosts of *M. enterolobii* in the US, are mostly grown in North Florida, and much less in South and Central Florida. Knowing the prevalence, hosts, and geographic distribution of *M. enterolobii* is extremely important to help limit its introduction and dissemination in new areas. Because of its broad host range and the lack of RKN resistance in commercially-available vegetable and fruit cultivars, current options for managing *M. enterolobii* are extremely limited, which is why increased awareness is so important. This is especially important considering that *M. enterolobii* has been included in the list of quarantinable pathogens in some states in the USA and Europe. Knowledge from this study on the prevalence, hosts, and distribution of the nematode in Florida is critical and can be used for improving and establishing new control measures for RKN, particularly *M. enterolobii*.
